# Predictors of Morbidity and Mortality After Colorectal Surgery in Patients With Cirrhotic Liver Disease–A Retrospective Analysis of 54 Cases at a Tertiary Care Center

**DOI:** 10.3389/fmed.2022.886566

**Published:** 2022-06-22

**Authors:** Cornelius J. van Beekum, Christina Beckmann, Alexander Semaan, Steffen Manekeller, Hanno Matthaei, Lara Braun, Maria A. Willis, Jörg C. Kalff, Tim O. Vilz

**Affiliations:** Department of General, Visceral, Thoracic and Vascular Surgery, University Hospital of Bonn, Bonn, Germany

**Keywords:** cirrhotic liver disease, colorectal surgery, morbidity, mortality, colon, rectum

## Abstract

**Background:**

Despite various existing scores that predict morbidity and mortality of patients with cirrhotic liver disease (CLD), data on specific risk stratification of patients with CLD undergoing colorectal surgery (CRS) are rare. The aim of this study was to assess in-hospital morbidity and mortality of patients with liver cirrhosis scheduled for CRS, with specific focus on possible pitfalls of surgery in this special cohort.

**Methods:**

Between 1996 and 2018, 54 patients with CLD undergoing CRS were identified and included in this study cohort. Postoperative morbidity and mortality were assessed using the Clavien/Dindo (C/D) classification as well as by type of complication. Univariate and multivariate analyses were performed to analyze the predictive factors for increased postoperative morbidity.

**Results:**

Of the patients, 37% patients died during the procedure or postoperatively. Major complications were seen in 23.1% of patients (>C/D IIIb). Patients with Child B or C cirrhosis as well as patients undergoing emergency surgery experienced significantly more major complications (*p* = 0.04 and *p* = 0.023, respectively). The most common complications were bleeding requiring blood transfusion (51.1%) and cardiocirculatory instability due to bleeding or sepsis (44.4%). In 53.7% of patients, an anastomosis was created without a protective ostomy. Anastomotic leakage occurred in 20.7% of these patients. Multivariate analysis showed that a primary anastomosis without a protective ostomy was the strongest risk factor for major complications (*p* = 0.042).

**Discussion:**

Morbidity and mortality after CRS in patients with CLD remains high and is not only influenced by liver function but also by surgical variables. Considering the high rate of anastomotic leakage, creating a protective or definitive ostomy must be considered with regard to the underlying pathology, the extent of CLD, and the patient's condition. Moreover, our data suggest that surgery in these most fragile patients should be performed only in experienced centers with immediate contact to hepatologists and experts in hemostasis.

## Introduction

Cirrhotic liver disease (CLD) is the common end stage of a plethora of chronic injuries to the liver such as viral hepatitis, non-alcoholic steatohepatitis, and alcoholic hepatitis. These pathogenic stimuli first result in structural changes with excessive accumulation of extracellular matrix proteins such as collagen (fibrosis). This results in the destruction of the cellular architecture of the liver (cirrhosis). Consequently, functional liver tissue is reduced with a consecutive insufficiency of normal function. With decreasing liver function, the reduction of renal function and pulmonary function can also be observed. Increasing stiffness of liver tissue causes portal hypertension with the development of collaterals to the mesentericoportal circulation and spontaneous portosystemic shunts (1). Data from the Global Burden of Disease study showed that the age-standardized incidence rate of CLD was 20.7 per 100,000 in 2015, a 13% increase from 2000, with men being affected significantly more often than women (2). Because of pathophysiologic changes in homeostasis, patients with CLD have a higher risk of morbidity and mortality following surgery.

Surgery of the colon and rectum is among the most common surgical procedures in daily practice. The evaluation of clinical fitness to undergo colorectal surgery (CRS) in pre-existing CLD remains a clinical dilemma. Complications of cirrhosis such as coagulopathy, portal hypertension with varices, and ascites increase the immediate surgical risk. Surgery strongly increases the risk of acute or chronic liver failure (ACLF) postoperatively (3–5). It has been shown that morbidity and mortality among patients with CLD undergoing surgery for CRS are higher during hospitalization and up to 30 days, 90 days, and 1 year postoperatively (6, 7). On the other hand, minimally invasive technique, a more elaborate, patient-oriented surgical approach, and improved medical management of liver cirrhosis, has improved the surgical outcomes in the past. Nevertheless, deciding whether patients with CLD are clinically ready to undergo major abdominal surgery necessitates a specific, surgical risk assessment, especially since surgical decision making in these cases becomes more frequent, with an increase in the proportion of patients with CLD undergoing CRS to 1 in 100 in the past decades (7). If surgery is inevitable, for instance because of colonic perforation, a better understanding of risk factors associated with postoperative morbidity and mortality depending on the severity of cirrhosis and severity of the abdominal catastrophe is desirable. Despite existing methods for staging general morbidity and mortality of patients with CLD (i.e., Child–Turcotte–Pugh [CTP] stage and Model of End-Stage Liver Disease [MELD] score), data on specific surgical risk assessment of patients with CLD undergoing CRS (CRS) remain scarce.

In this study, we analyzed intraoperative and postoperative variables in patients after CRS and macroscopically or histologically confirmed liver cirrhosis. The aim of the study was to identify potentially modifiable risk factors to optimize the patient's condition prior to surgery and identify pitfalls of CRS in this special patient group to reduce postoperative morbidity and mortality.

## Patients and Methods

### Patient Selection

**Figure 1:**
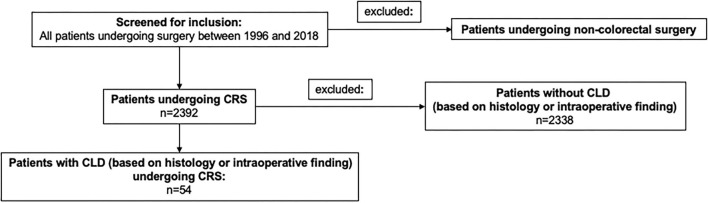
Flow chart on patient selection and exclusion.

We retrospectively identified all patients who subsequently underwent CRS between 1 January 1996 and 31 December 2018 at the University Hospital of Bonn, Germany. Only patients with a CLD diagnosis based on histological examination or intraoperative findings were included in this study (Figure 1). Data were obtained from the patients' medical charts, discharge letters, surgical reports, and anesthesiologic protocols. Demographics and laboratory data, medical or interventional therapy, and histological reports were analyzed.

### Surgery

All patients underwent surgery of the colon and rectum. Procedures included right hemicolectomy, left hemicolectomy, resection of the transverse colon, sigmoid resection, subtotal, or total colectomy, proctocolectomy, colostomy, and reversal of Hartmann's procedure. Few patients received minor additional surgery (hernia repair, biopsy of the liver).

### Severeness of Liver Cirrhosis

To determine severeness of CLD, CTP stage, and MELD scores were calculated.

### Morbidity and Mortality

The Dindo/Clavien (D/C) score was used to classify the postoperative complications. Severe complications were defined as D/C grade ≥ IIIB (8). Morbidity was further categorized as follows:

- Cardiocirculatory instability requiring administration of vasopressors (caused by various reasons, i.e., bleeding, sepsis, and cardiac shock)
- Bleeding requiring transfusion of two or more units of red blood cells
- Surgical site infection
- Peritonitis
- Respiratory complications (necessity of thoracocentesis and mechanical ventilation)
- Renal complications (renal replacement therapy)
- Hydropic decompensation (necessity of abdominocentesis)
- Anastomotic leakage
- Infectious complications (other than surgical site infection).

### Statistical Analysis

The patient data were evaluated using the IBM SPSS Statistics computer program (Version 25.0, IBM Corporation, Armonk, USA). The level of significance was chosen as α = 0.05.

For the statistical description of the data, the frequency, the arithmetic mean, the median, the range with minimum and maximum, the standard deviation, and *p*-values were determined and are presented below. Various statistical tests were used to determine the influencing factors. For the analysis of variance, the Fisher test was used. The test for exact significance was carried out on both sides. The Cochran–Armitage trend test was used to check whether several variables can be viewed as varying linearly. The ranks formed were tested for significance by the Mann–Whitney *U* test. Since there were more than 30 patients, the asymptotic significance was calculated. Spearman's rank correlation and chi-square tests were used. In addition, a univariate regression analysis was carried out to test relationships, the significant values of which were finally analyzed in a multivariate model.

## Results

### Patient Characteristics

Of all patients who underwent CRS between 1 January 1996 and 31 December 2018 at the University Hospital of Bonn, Germany; 54 patients with CLD diagnosis based on histological examination or intraoperative findings were included in this study and further analyzed.

Of the patients, 42 (77.8%) patients were male and the median age was 61.5 years (30–90 years). The median body mass index (BMI) was 25.4 (17.7–35.4). The morbid obesity was found in 14 patients (25.9%). Most of patients had alcoholic CLD (42.6%), followed by 29.6% of patients in whom no underlying cause of cirrhosis was found. Considering the high prevalence of diabetes and obesity in our cohort, a relevant number of these patients defined as cryptogenic conceivably “suffered” from nonalcoholic steatohepatitis (NASH). Among the 29.6% patients with cryptogenic cirrhosis, five showed BMI >30 (9.3%) and most likely suffered from NASH cirrhosis. Median MELD was 9 while over half (53.7%) of the patients exhibited CTP A cirrhosis. Median albumin was 3.1 mg/dl (interquartile range [*IQR*] 1.0–4.0) (Table 1). Regarding preoperative physical status classification (American Society of Anesthesiologists [ASA] score), 48.1% (*n* = 26) of patients were considered ASA III, 27.8% (*n* = 15) of patients were designated ASA II, and 7.4% (*n* = 4) of patients considered ASA IV, and 16.7% (*n* = 9) of patients were classified as ASA V. Of note, only one of the ASA V patients survived the procedure (Table 2).

**Table 1:** Patient characteristics.

**Characteristics**	**Mean (SD)/f (f %)**	**Median**	**Min.-Max**.
**Age (years)**	61.0 ± 13.5	61.5	30–90
**Sex**			
Male	42 (77.8 %)		
Female	12 (22.2 %)		
**Height (cm)**	174.1 ± 8.9	173	156–193
**Weight (kg)**	81.3 ± 19.4	80	48–130
**BMI**	26.4 kg/m^2^ ± 4.9	25.4	17.7–35.4
Alcoholic	23 (42.6 %)		
Cryptogenic (inter alia, NASH)	16 (29.6 %)		
Viral	6 (11.1 %)		
PBC	6 (11.1 %)		
Autoimmune hepatitis	1 (1.9 %)		
Other	2 (3.7 %)		
**Child-Turcotte-Pugh stage**			
A	29 (53.7 %)		
B	14 (25.9 %)		
C	6 (11.1 %)		
**MELD score**	12.3 ± 6.9	9	6–31
**Laboratory values**			
INR	1.2 ± 0.4	1.10	0.9–3.8
Bilirubin (mg/dl)	1.7 ± 2.4	0.92	0.2–10.8
Creatinine (mg/dl)	1.7 ± 1.7	1.0	0.4–9.0
Albumin (g/dl)	3.0 ± 0.7	3.1	1.0–4.0
Thrombocytes (G/l)	220.6 ± 111.0	223	24–439
**Ascites**			
No/little	30 (55.6 %)		
Moderate/treatable	9 (16.7 %)		
Massive/refractory	5 (9.3 %)		
**Portal hypertension**	19 (35.2 %)		
**Esophageal varices**	15 (27.8 %)		
**Splenomegaly**	14 (25.9 %)		
**Icterus**	6 (11.1 %)		
**Hepatocellular carcinoma**	4 (7.4 %)		
**Encephalopathy**	3 (5.6 %)		

*PBC, Primary biliary cholangitis; MELD, Model-of-end-stage-liver-disease; INR, International Normalized Ratio; SD, standard deviation; f, frequency; f %, frequency in percent; N = 54*.

**Table 2:** Surgical variables.

**Characteristics**	**Mean (SD) /**	**Median**	**Min.-**
	**f (f %)**		**Max**.
**Procedure**			
Right hemicolectomy	16 (29.6 %)		
Left hemicolectomy	8 (14.8 %)		
Resection of the transverse colon	5 (9.3 %)		
Colectomy	4 (7.4 %)		
Sigmoid resection	15 (27.8 %)		
Reversal of Hartmann's procedure	2 (3.7 %)		
Colostomy	4 (7.4 %)		
**Postoperative intestinal continuity**			
Primary anastomosis	29 (53.7 %)		
Hartmann's procedure	17 (31.5 %)		
Diverting ostomy	8 (14.8 %)		
**Surgical approach**			
Open	48 (88.9 %)		
Laparoscopic	4 (7.4 %)		
Conversion	2 (3.7 %)		
**Urgency**			
Emergency	21 (38.9 %)		
Elective	33 (61.1 %)		
**ASA classification**			
1	0 (0 %)		
2	15 (27.8 %)		
3	26 (48.1 %)		
4	4 (7.4 %)		
5	9 (16.7 %)		

*ASA, American Society of Anesthesiologists; SD, standard deviation; f, frequency; f %, frequency in percent; N = 54*.

Of the patients, 25 (50%) patients suffered from prior cardiac disorders (atrial fibrillation, coronary heart disease, chronic heart failure, and prior myocardial infarction), 22 (40.7%) patients suffered from acute or chronic renal failure, 11 (20.4%) patients suffered from prior stroke or epilepsy, and 10 (18.5%) patients suffered from diabetes mellitus or obesity (BMI > 30 kg/m^2^). Pulmonary comorbidities (e.g., chronic obstructive pulmonary disease) were seen in 19 patients (35.2%) and peripheral arterial disease and other atherosclerotic diseases were seen in 25 patients (46.3%) (Table 3). The most common comorbidity was arterial hypertension (*n* = 32, 59.3%). No a single patient showed signs of acute or chronic liver failure prior to surgery.

**Table 3:** Comorbidities.

	**f (f %)**
Arterial hypertension	32 (59.3 %)
Cardiac CM	27 (50.0 %)
Vascular CM	25 (46.3 %)
Renal CM	22 (40.7 %)
Pulmonal CM	19 (35.2 %)
Neurological CM	11 (20.4 %)
Diabetes mellitus	10 (18.5 %)

*CM, Comorbidity; f, frequency; f %, frequency in percent; N = 54*.

### Surgical Therapy

Surgical variables are presented in Table 2. Right-sided hemicolectomy (29.6%) and resection of the sigmoid colon (27.8%) were the most common procedures. In 53.7% (*n* = 29) of the procedures, a primary anastomosis without a protective ostomy was performed, while 14.8% of patients received a diverting loop ileostomy. Most cases were performed with an open surgical approach (88.9%). In two patients, a primary laparoscopic approach was converted to an open laparotomy (3.7%). Of the procedures, 61.1% (*n* = 33) were elective and 38.9% (*n* = 21) were in an emergency setting. In addition to CRS, 13% (*n* = 7) patients received a cholecystectomy, and hernia repair was needed in 7.4% (*n* = 4) of all surgeries. Liver biopsy was performed in 59.3% (*n* = 32) of patients.

#### Indications for Surgery

Cancers of the colon or rectum (*n* = 24, 44.4%), colon perforation (*n* = 10, 18.5%), and diverticulitis without perforation (*n* = 9, 16.7%) were the most common indications for surgery. Surgery was also performed because of ischemia (*n* = 4, 7.4%), bleeding (*n* = 2, 3.7%), stenosis of a pre-existing ostomy (*n* = 2, 3.7%), and reversal of Hartmann's procedure (*n* = 2, 3.7%).

### Intraoperative Transfusion of Blood Products

Transfusion of red blood cells was needed in 22 patients during the procedure, with a maximum of 16 units of red blood cells. There were 32 patients who received fresh frozen plasma, with a maximum of 12 units. Using Fisher's test, no significant correlation was found between necessity of transfusion and emergency surgery. The Cochran–Armitage trend test showed no significant trend concerning transfusion of red blood cells and postoperative major complications (>D/C IIIb) in contrast to transfusion of plasma, where a significant trend toward major complications was found (*p* = 0.031^*^). Interestingly, using Spearman's rank correlation, a statistically significant interdependence between the number of red blood cell-units and units of plasma and severity of postoperative complications was found (*p* = 0.04^*^ and *p* = 0.036^*^, respectively).

### Postoperative Complications

#### General Post-operative Complications

More than half of patients experienced major complications (> D/C IIIb) (59.3%, *n* = 22). Of the 54 included patients 20 (37%) died intraoperatively or in the postoperative course (D/C V, Table 4). In detail, seven patients died on table, five during Hartmann's procedure, and 13 patients died in the postoperative course. In 15 patients, redo procedures were necessary (27.8%). A significantly higher risk of redo procedures was observed after emergency CRS (*p* = 0.013^*^). In 44.4% of cases (*n* = 24) cardiocirculatory instability requiring vasopressor therapy occurred, with bleeding (*n* = 21, 38.9%), surgical site infection (*n* = 20, 37%), and peritonitis (*n* = 17, 31.5%) being the most common accompanying complications (Table 5).

**Table 4:** Complications according to Dindo/Clavien.

**D/C classification**	**f (f %)**
I	5 (9.3 %)
II	15 (27.8 %)
IIIa	2 (3.7 %)
IIIb	11 (20.4 %)
IV	1 (1.9 %)
V	20 (37 %)

*D/C, Dindo/Clavien; f, frequency; f %, frequency in percent; N = 54*.

**Table 5:** Post-operative complications.

	**f (f %)**
Cardiocirculatory instability	24 (44.4 %)
Bleeding	21 (38.9 %)
Surgical site infections	20 (37.0 %)
Peritonitis	17 (31.5 %)
Respiratory complications	16 (29.6 %)
Renal complications	16 (29.6 %)
Hydropic decompensation	13 (24.1 %)
Anastomotic leakage	6 (11.1 %)
**Infection**	
Urinary tract	5 (9.3 %)
Pneumonia	5 (9.3 %)
Other	15 (27.8 %)

*f, frequency; f %, frequency in percent; N = 54*.

Severity of preoperative presence of ascites correlated with morbidity according to Spearman's rank correlation (*p* = 0.024^*^). Decompensated ascites was seen in 13 patients (24.1%) postoperatively, although no correlation with a higher-grade morbidity was found. Postoperative peritonitis was also significantly correlated with severity of postoperative complications using the chi-square test (*p* = 0.038^*^).

#### Anastomotic Leakage and Mortality

Anastomotic leakage occurred in six of the 29 cases in which a primary anastomosis was created (20.7%), and leakage of the colonic or rectal stump after Hartmann's procedure was observed in two of 17 cases (11.8%). Of the 29 patients receiving a primary anastomosis, nine (31%) died (Table 6).

**Table 6:** Mortality and rate of anastomotic leakage.

	**Total**	**Mortality**	**Insufficiency**
Primary anastomosis	29	9	6
Hartmann's procedure	17	8	2
Diverting ostomy	8	3	0

#### Univariate Analysis of Post-operative Morbidity and Mortality

Univariate regression analysis showed significant correlation of ASA status and major complications as well as death (*p* = 0.022^*^ and *p* = 0.000^*^, respectively). In contrast to procedures performed in an elective setting, emergency surgery was accompanied by an increased risk of major complications and mortality (*p* = 0.003^*^ and *p* = 0.008^*^, respectively). The CTP stage showed significant correlation with the occurrence of major complications and mortality in univariate regression analysis (*p* = 0.038^*^ and 0.006^*^, respectively). The probability of mortality increases with CTP stage: 7% of patients in CTP stage A died, 14% in CTP B, and 67% in CTP C. This finding was also observed using the MELD score (Table 7). While morbidity and mortality significantly increased with higher CTP stage and MELD scores, using the Mann–Whitney *U* test, no correlation between CTP stage and MELD score and the probability of anastomotic leakage was found (CTP: *p* = 0.888; MELD: *p* = 0.435).

**Table 7:** Univariate analysis of risk factors indicating postoperative morbidity.

	***p***	**Odds ratio**	**95% confidence interval**
CTP value	0.038^*^	1.633	1.028–2.594
ASA classification	0.022^*^	2.237	1.125–4.452
MELD Score	0.034^*^	1.112	1.008–1.228
Emergency procedure	0.003^*^	8.143	2.001–33.144
Albumin value	0.027^*^	0.269	0.084–0.861
Malignant disease	0.021^*^	0.260	0.083 −0.817
Primary anastomosis	0.006^*^	5.667	0.052–0.602
Hartmann's procedure	0.026^*^	4.926	1.210–20.050

Significance at 5% level/* = p <0.05. N = 54.

When investigating laboratory values, only low albumin was significantly correlated with an increased risk of morbidity (*p* = 0.027^*^) but not mortality. Low platelets, low Internationally Normalized Ratio (INR), and bilirubin were not correlated with increased morbidity or mortality.

Although surgery for malignant disease increased the risk of major complications in the postoperative course (*p* = 0.021^*^), surgery in presence of hepatocellular carcinoma did not (*p* = 0.182).

Only pre-existing renal dysfunction (not creatinine value alone) significantly predicted morbidity in univariate regression analysis (*p* = 0.043^*^).

Interestingly, factors such as age, morbid obesity, pre-existing diabetes, and intraoperative transfusion did not significantly predict morbidity or mortality in univariate regression analysis.

#### Multivariate Analysis of Post-operative Morbidity and Mortality

All variables significantly predicting morbidity in univariate regression analysis were considered for multivariate regression analysis. Here, only a primary anastomosis without diverting ostomy was associated with increased morbidity (*p* = 0.013^*^).

## Discussion

Cirrhotic liver disease is a known major risk factor for perioperative morbidity and mortality after surgery in general and much so after CRS. In patients undergoing non-transplant surgery, the usual scoring systems determining the severity of cirrhosis are correlated with increased postoperative morbidity, i.e., CTP stage in estimating 30-day morbidity and MELD for estimating mortality within the first 3 months postoperatively (9). Our data underline this finding, demonstrating increased mortality with increased CTP stage and a higher morbidity depending on both, CTP stage and MELD scores. Meunier et al. reported a 26% mortality in 41 patients with CLD undergoing CRS for various indications (10). Our data exceeded this already high mortality, showing 37% in-hospital mortality irrespective of an elective or emergency setting. The results of this study correlate well with the data of Nguyen et al. showing a 35.8% mortality in a cohort of a nationwide inpatient sample with cirrhosis and portal hypertension undergoing emergency CRS (11). Indeed, our data as well suggest a strong trend toward morbidity and mortality depending on the acuteness of surgery and CTP/MELD stage.

Several parameters related to the acuteness of surgery and CTP and MELD stages have been evaluated as possible predictors of morbidity and mortality in CLD patients undergoing CRS. The previous studies have identified hepatic coagulopathy complicating surgery in this patient group (12). Indeed, we were able to determine a 38.9% bleeding complication rate in our cohort although altered standard coagulation laboratory tests did not predict morbidity or mortality. Interestingly, in a previous study of ours, hemorrhage was the most common complication after small bowel surgery in presence of CLD, although no correlation to altered coagulation parameters or stage of liver disease (13). This agrees with clinical experience that because of the patients' altered intra-abdominal hemodynamic balance and hepatic coagulopathy but normal classical laboratory tests (INR and/or prothrombin time), patients with CLD are at a much higher risk for relevant hemorrhage. Hepatic insufficiency alters pro- and anti-hemostatic pathways, providing a fragile balance to the patient's coagulation status, which is additionally unbalanced by an operative trauma including, for example, hypothermia (14). Contrary to our findings, prothrombin time prolongation predicted morbidity in a multivariate analysis of 161 patients with CLD undergoing CRS (15), stressing that a thorough examination of the patient's hemostatic situation is particularly desirable. Planning any surgical intervention must, therefore, include close consultation of hemostasis experts and a comprehensive laboratory workup (i.e., repetitive global coagulation tests) seems advisable.

Low albumin presented as a significant predictor of postoperative morbidity in our cohort. Preoperative low albumin has been identified as an independent risk factor for postoperative morbidity and mortality after CRS irrespective of a pre-existing liver disease even in highly advanced surgery within enhanced recovery pathways (16, 17). Low albumin reflects on poorer nutritional status and immune functions in CLD patients. Different scores and ratios consisting of albumin, platelets, hemoglobin, and lymphocytes representing the inflammatory status of the patient prior to surgery have been evaluated and correlated with morbidity and mortality in one form or another (18–20). In general, an equation of preoperative immune status and nutritional status seems to correlate with postoperative outcome and potentially even long-term oncological survival. Yet final validation of these scores and introduction into clinical practice are still absent. In patients with CLD, decreased hepatic synthesis results in altered immune function and malnutrition. Malnutrition is commonly seen in patients with cirrhosis with an incidence of 60–100% in patients with advanced CLD. Malnutrition is a poor prognostic factor associated with lower survival and morbidity in patients with CLD even without a traumatic stimulus such as abdominal surgery but especially thereafter (21–26). Cirrhosis-associated immune dysfunction describes an increased inflammatory phenotype of systemic immune cells with a decreased antipathogenic functionality, resulting in infectious and inflammatory complications. This becomes especially relevant in patients with intestinal dysbiosis due to therapeutic interventions let alone antibiotic therapy. In this context, the gut–liver–immune axis has become an important target in therapy of CLD. Alterations of this axis by surgical manipulation of the intestine lead to alterations in immune homeostasis and potentially decrease residual liver function (27). In our cohort, surgical site infection and peritonitis were two of the most common complications following CRS in patients with CLD, reflecting on the patients' altered immune response and highlighting the importance of preoperative optimization of the functional and nutritional status of patients with CLD.

The most interesting result of our study is the significantly higher rate of major complications and thus morbidity when a primary anastomosis without a protective ostomy was created. The rate of anastomotic leakage, the most catastrophic complication in our cohort, was very high, 20.7%, leading to a higher overall morbidity and mortality. CLD has not yet been clearly identified as a risk factor for anastomotic leakage in the recent literature (28, 29). In detail, a study by Sabbagh et al. (27) showed no differences in anastomotic healing in 40 patients with CLD compared to 80 non-CLD patients. Of note, the acuteness of surgery and Child stage of patients must be taken into consideration when comparing the results of these studies. To our knowledge, only one study stratified the higher risk of anastomotic leakage in the presence of CLD (30). In a retrospective analysis of patients of a prospective colorectal database, Käser et al. showed a 12.5% rate of anastomotic leakage in patients with CLD compared to a 2.5% rate of anastomotic leakage in non-CLD patients. Arguably, with only 24 patients in the CLD cohort *vs*. 1,851 patients in the non-CLD cohort, the sample size was rather small and underlying reasons for defective anastomotic healing were not given. Altered intra-abdominal hemodynamics due to portosystemic collaterals, ascites, intestinal dysbiosis, and altered immune response can be responsible for this finding (13, 31). In an animal model of anastomotic healing in rats with CLD, a decreased concentration of hydroxyproline was found in anastomotic regions of cirrhotic rats compared to healthy controls. Since hydroxyproline is important in stabilizing the collagen in the anastomotic junction, this finding should be evaluated in further studies (32). Malnutrition and low protein levels have been evaluated as an influence in animal studies as well (33). It has also been shown in a retrospective study in humans that low albumin is associated with disturbances in anastomotic healing (34). It might be hypothesized that in our cohort, a primary anastomosis was only created in patients with mild cirrhosis. Still, these patients were more likely to develop severe complications, with a mortality of 31%. In accord with the literature, low albumin and intestinal dysbiosis with an altered gut–immune–liver axis as a marker of liver dysfunction could be associated with the poor outcomes after CRS (35, 36). Additionally, in our cohort, a considerable number of patients (18.5%) suffered from diabetes, which is also a risk factor for anastomotic leakage and should be taken into consideration. A retrospective analysis of patients undergoing anterior rectal resection showed an incidence of anastomotic leakage of 9.3% in patients with diabetes (odds ratio = 2.906, 95% confidence interval 1.130–7.475; *p* = 0.027) (37). In our cohort, the incidence of anastomotic leakage was higher with 20.7%. Furthermore, it was not investigated whether patients continuously consumed alcohol or had already stopped drinking before surgery. It is hypothesized that continued alcohol abuse itself is a risk factor for anastomotic leakage. An incidence of anastomotic leakage of 21.3% was described in patients with continued alcohol abuse (38), although alcohol consumption was not associated with leakage in a large meta-analysis of 17 studies (39). Pathologic abuse of alcohol might be a surrogate for poor nutritional status and decreased liver function with consecutively decreased the protein status in these patients, even if no manifest CLD has developed yet. In this context reasons for anastomotic leakage in cirrhotic patients and patients with continued alcoholism are multifactorial (portal hypertension with impaired regulation of splanchnic blood flow, protein metabolism disorder, immune dysfunction syndrome especially in the presence of ascites). When investigating anastomotic leakage in patients with CLD specifically, protein status, presence of ascites, peritonitis, acuteness of surgery, requirement for vasopressor therapy, etc., must be considered and should be analyzed in further upcoming studies.

It should be mentioned that the performance of Hartmann's procedure was also predictive of a higher morbidity. Arguably, there might be a bias here as discontinuing resection of the colon was performed only in high-risk patients with severe cirrhosis susceptible to a higher complication rate. Interestingly, no correlation between CTP and MELD stage and the occurrence of anastomotic leakage was found in our cohort, suggesting that anastomotic leakage, the most feared complication after CRS, can occur at any stage of cirrhosis with fatal consequences.

Because of the high rate of anastomotic leakage in our cohort, the creation of a protective ostomy must be considered in patients with liver cirrhosis. Of note, various factors such as pre-existing diabetes, continued alcohol abuse, and the presence of peritonitis and ascites must be taken into consideration when deciding whether to create a primary anastomosis in patients with CLD. Interestingly, CTP stage and MELD score did not influence the probability of anastomotic leakage in our cohort, although this might be attributed to the small sample size.

Limitations of this study are its retrospective nature with a relatively small sample size and thus an obvious lack of statistical power. Interestingly, this is a problem of most studies investigating the postoperative outcomes in patients with CLD, with the most recent study investigating the colectomy in patients with CLD identifying only 248 patients with CLD in a cohort of 36,380 patients after CRS (7). However, we tried to reduce any bias by including all consecutive patients at a single tertiary referral center who underwent CRS with or without anastomosis. Despite these limitations, our study delivered valid results to be considered by colorectal surgeons, especially concerning the risk of anastomotic leakage. Potential preoperative optimization of functional and especially nutritional status should be considered and further investigated in prospective studies concerning surgery in patients with CLD.

## Conclusions

Morbidity and mortality after CRS in patients with CLD remain high and are influenced not only by liver function but also by surgical variables. Considering the high rate of anastomotic leakage, construction of a protective or definitive ostomy must be considered, taking pre- and intraoperative variables into account. Preoperative optimization of patients' functional and nutritional status should be considered if possible. Moreover, our data suggest that surgery in these most fragile patients should be performed only in experienced centers with immediate contact to medical specialists and experts in hemostasis.

## Data Availability Statement

The raw data supporting the conclusions of this article will be made available by the authors, without undue reservation.

## Ethics Statement

Ethical review and approval was not required for the study on human participants in accordance with the local legislation and institutional requirements. Written informed consent for participation was not required for this study in accordance with the national legislation and the institutional requirements.

## Author Contributions

Conceptualization, data curation, formal analysis, investigation, and writing–original draft: CVB, TV, and CB. Methodology: CVB, LB, MW, CB, and TV. Resources: JK. Supervision: HM, SM, and JK. Writing–review and editing: CVB, AS, HM, SM, LB, MW, JK, and TV. All authors have read and agreed to the published version of the manuscript.

## Conflict of Interest

The authors declare that the research was conducted in the absence of any commercial or financial relationships that could be construed as a potential conflict of interest.

## Publisher's Note

All claims expressed in this article are solely those of the authors and do not necessarily represent those of their affiliated organizations, or those of the publisher, the editors and the reviewers. Any product that may be evaluated in this article, or claim that may be made by its manufacturer, is not guaranteed or endorsed by the publisher.
